# Synthesis and Characterization of [Fe(Htrz)_2_(trz)](BF_4_)] Nanocubes

**DOI:** 10.3390/molecules27041213

**Published:** 2022-02-11

**Authors:** Alexis A. Blanco, Daniel J. Adams, Jason D. Azoulay, Leonard Spinu, John B. Wiley

**Affiliations:** 1 Departments of Chemistry and Physics, Advanced Materials Research Institute, University of New Orleans, New Orleans, LA 70148, USA; aablanc1@my.uno.edu (A.A.B.); lspinu@uno.edu (L.S.); 2 School of Polymer Science and Engineering, The University of Southern Mississippi, Hattiesburg, MS 39406, USA; daniel.adams@usm.edu (D.J.A.); jason.azoulay@usm.edu (J.D.A.)

**Keywords:** spin crossover, Tergitol NP9, size study, nanoparticles, thermal hysteresis, transmission electron microscope, SQUID, reverse micelle

## Abstract

Compounds that exhibit spin-crossover (SCO) type behavior have been extensively investigated due to their ability to act as molecular switches. Depending on the coordinating ligand, in this case *1H*-1,2,4-triazole, and the crystallite size of the SCO compound produced, the energy requirement for the spin state transition can vary. Here, SCO [Fe(Htrz)_2_(trz)](BF_4_)] nanoparticles were synthesized using modified reverse micelle methods. Reaction conditions and reagent ratios are strictly controlled to produce nanocubes of 40–50 nm in size. Decreases in energy requirements are seen in both thermal and magnetic transitions for the smaller sized crystallites, where, compared to bulk materials, a decrease of as much as 20 °C can be seen in low to high spin state transitions.

## 1. Introduction

The spin transition phenomenon known as spin-crossover (SCO) was originally observed more than 85 years ago [1]. Since that time, there have been numerous studies on compounds that undergo this spin transition phenomenon [2,3,4,5,6,7,8,9,10,11,12,13,14]. A spin transition is an example of electronic bistability at the molecular level impacted by the coordinating ligands. The ability for a compound to be stable in two different states makes an ideal molecular switch and is attractive for potential use in various devices such as displays [12], data storage units [10], mechanical actuators [5], and optical sensors [15]. In the field of SCO materials, an iron(II) triazole complex, [Fe(Htrz)_2_(trz)](BF_4_)] (Htrz = 1H-1,2,4-riazole and trz = deprotonated triazolato(–) ligand), has been touted as the best for such applications due to its wide hysteretic behavior above room temperature.

**Figure 1:**
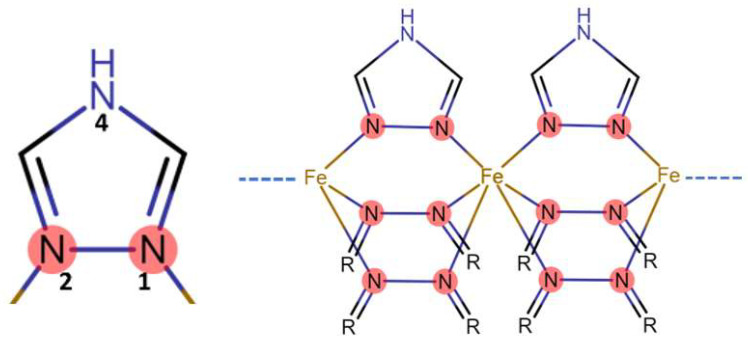
Left, triazole ligand with nitrogens at the 1, 2, and 4 positions. Right, triazole complex showing the N^1^, N^2^ triazole triple bridge that supports the 1D coordination chains of the structure. (R is used to simplify image, and represents bridging nitrogen (4) as shown in the 1,2,4-triazole ligand on the left.)

This bistable SCO compound [Fe(Htrz)_2_(trz)](BF_4_)] can exist in two interchangeable states depending on the external stimuli. The electronic configurations of the Fe(II) in this system can be switched between low spin and high spin states, using stimuli such as temperature [16], irradiation [16], and magnetic fields [8]. This low to high spin transition or vice versa impacts various properties within the material, including optical, magnetic, mechanical, and electrical characteristics [10]. The transition in Fe(II) compounds is associated with diamagnetic to paramagnetic switching, as well [16]. Triazole ligands provide ligand-field strengths around Fe(II) ions that are sufficient for the occurrence of SCO [17,18,19,20]. These ligands can also provide chemical flexibility while supporting the 1D coordination chains of Fe(II) through triple N^1^, N^2^-triazole bridges (Figure 1) [15]. It is this short, rigid strain-less system that allows triazole 1D SCO Fe(II) systems to be quite stable in both the low and high spin states. Bulk SCO materials are typically insulating, and their electrical properties were ignored until recently with the emergence of nanoscale SCO materials [21,22].

The majority of Fe(II) SCO compounds have been studied in the bulk level (large micron-sized crystals). There has now been a push to investigate these systems at the nanoscale to determine whether significant property changes occur relative to the bulk. Attempts at fabricating SCO nanoparticles have been performed using triazole based coordination polymers via a reverse micelle technique [23,24]. Forestier et al. used [Fe-(atrz)_3_]^2+^ derivatives (atrz = amino triazole) and Lauropal as the surfactant, producing 69 nm spherical particles [23], while Coronado et al. fabricated [Fe(Htrz)_2_(trz)](BF_4_)] spherical 15 nm particles using sodium dioctyl sulfosuccinate [24]. Both systems showed slightly reduced variations in spin transition temperatures and hysteresis widths when compared to bulk materials. Simulations have suggested that 10 nm is the smallest nanoparticles size for which a cooperative SCO can be seen [25]. One important concern for the smaller sizes is that, as particles continue to shrink in size, greater aggregation tends to occur. This may strongly influence the magnetic properties of the nanoscale systems [8].

Herein, we investigate the synthesis of [Fe(Htrz)_2_(trz)](BF_4_)] nanoparticles via a reverse micelle synthesis using Tergitol NP9 (Nonylphenol Ethoxylate) as the surfactant. Tergitol NP9 was necessary to control of the size of [Fe(Htrz)_2_(trz)](BF_4_)] nanocubes, producing particles with an average size of 49 nm. Both thermal and magnetic analysis provided insightful data on the spin transition temperatures, which occurred around 100 °C for low spin to high spin and 65 °C for high spin to low spin transitions. These spin transitions occur at lower temperatures when compared to bulk analogs [8,11,14] and other nanoparticles made with different starting materials [23,24]. 

## 2. Results

**Figure 2:**
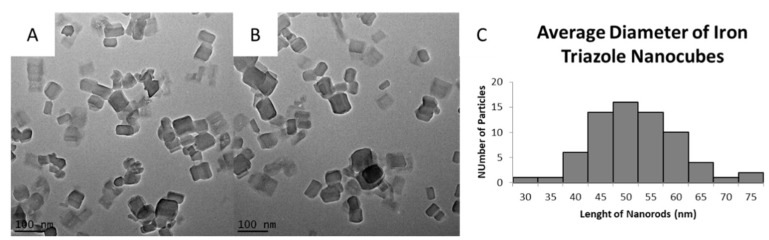
[Fe(Htrz)_2_(trz)](BF_4_)] nanocubes synthesized using the optimal method developed by adaptation of a known reverse micelle approach. (**A**,**B**) are from the same sample and separate cubic particles can be seen, along with some agglomerated particles. (**C**) is the particle’s distribution for the average diameter of the nanoparticles obtained in (**A**,**B**).

Synthesis. Adaptation of the reverse micelle methods using Tergitol NP9 as the surfactant and ascorbic acid as a mild reducing agent led to the formation of cubic nanoparticles of [Fe(Htrz)_2_(trz)](BF_4_)], with an average size of 49 ± 4 nm (Figure 2). In addition to the dominant cubic shape of the particles, other elongated and rounded shaped particles were also observed with TEM imaging, Figure 2A,B.

To optimize the synthesis of the [Fe(Htrz)_2_(trz)](BF_4_)] nanocubes, a variety of parameters were studied. Table S1 highlights the systematic approach that was used to achieve optimal conditions for the growth of the cubic structures. Adjustments to the reaction time, temperature, and surfactant ratio impacted size and morphology of the nanoparticles. If the system exceeds the optimal conditions to produce nanocubes (Reaction 9), nanorods are formed at varied lengths (Reactions 1–8). TEM images of nanorods obtained can be seen in Figure S1.

**Figure 3:**
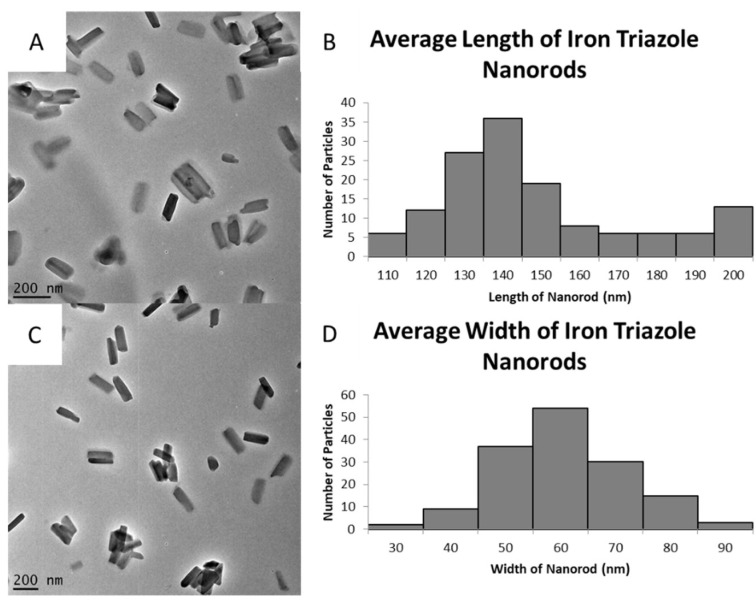
TEM images of particles that were obtained when precursors were separated within different vials and stirred for 5 min, Reaction 5. Both (**A**,**C**) are samples that were conducted with the same reaction conditions. (**B**,**D**) are average lengths and widths, respectively.

The role of the surfactant was found to be critical in directing the morphology and size of [Fe(Htrz)_2_(trz)](BF_4_)] nanoparticles. Initial reaction conditions, with a 24-h reaction time and no surfactant, led to samples dominated by large nanorods (average 476 ± 65 nm by 135 ± 33 nm) along with other particles of various morphologies and degrees of agglomeration (Figure S1A). Smaller nanorods, with average lengths of 309 ± 44 nm and widths of 45 ± 12 nm (Figure S1D), were obtained with a reaction time of 24-h with Tergitol NP9 surfactant and ascorbic acid. Considering the length of nanorods produced by the 24-h synthesis, systematic changes were made to the reaction time and the amount of ascorbic acid so as to target smaller particles. A reaction time of 5 min with 10 mg ascorbic acid (reduced from 50 mg) produced nanorods that had a wide variety of sizes, ranging from 73 to 800 nm in length (Figure S1B). Further modification of the reaction involved the use of a preliminary stirring step (5 min) prior to the combination of both starting materials. The resulting particles showed much greater uniformity and smaller sizes, with average lengths of 150 ± 31 nm and widths of 56 ± 12 nm. Figure 3 shows the TEM images and particle distribution analysis of these smaller particles using this preliminary stirring.

Given that Tergitol NP9 reduced the size of the particles by 70%, modification of the surfactant ratio was then conducted to determine if even smaller particles were possible. Original surfactant requirements specified a fixed surfactant amount of 75% (4 g) of the starting material; this was adjusted to either 80% (5 g) or 90% (10 g). Particles that were made with an 80% fixed surfactant amount showed much larger sizes (average lengths of 197 ± 44 nm) than those made with 75%. Using a fixed amount of 90% of the surfactant led to the production of partially formed nanoparticles. Throughout Reactions 2–5, ascorbic acid amounts were kept constant at 10 mg. This amount was then varied to determine any relationship to size control. Using less than 5 mg of ascorbic acid led to the formation of clumps of irregular particles, Figure S1C.

Based on all the previous modifications performed in Reactions 1–8, a further heating phase was implemented. Reaction 9 in Table S1 shows the optimal conditions that were used to synthesize [Fe(Htrz)_2_(trz)](BF_4_)] nanocubes. Tergitol NP9 was kept constant at 75% excess of the solvent amount, 1 mL of water as the solvent, and the precursors, Fe(BF_4_)_2_·6H_2_O and 1,2,4-triazole, were in a molar ratio of 1:3, respectively. The amount of ascorbic acid used was set at 5 mg based on the outcomes of Reactions 5 and 6, Figure 3A and Figure S1C, respectively. Heating at 80 °C and stirring of separate vials for 15 min before combination in a 1-h reaction time proved to be beneficial to the overall synthesis of the nanocubes.

**Figure 4:**
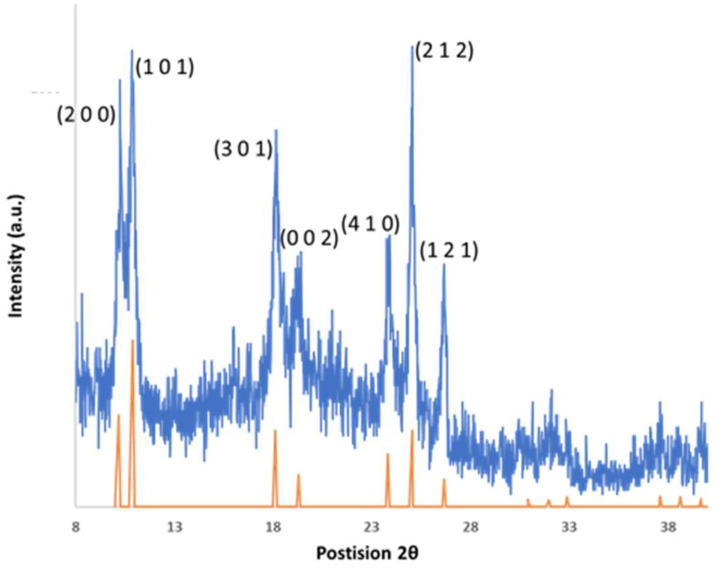
Observed XRD pattern for [Fe(Htrz)_2_(trz)](BF_4_)] (top, blue) versus the simulated low spin reference pattern (bottom, orange). Broad diffraction peaks are observed, consistent with the nanoparticle nature of the sample. Reference pattern calculated from published data [14].

Diffraction analysis. Simulated diffraction data were used as a reference for the peak positions of the observed XRD patterns of [Fe(Htrz)_2_(trz)](BF_4_)] [14]. Simulated patterns for both low and high spins are shown in Figure S2. There is a volume expansion in transitioning from the low to high spin state, with corresponding shifts in diffraction peaks. Observed XRD data for [Fe(Htrz)_2_(trz)](BF_4_)] are shown in Figure 4 and are in good agreement with the low spin structure. The distinguishable peaks, (2 0 0), (1 0 1), (3 0 1), (0 0 2), (4 1 0), (2 1 2), and (1 2 1), remained the same in each of the samples independent of reaction conditions. While bulk samples exhibit sharp reflections, nanoparticle samples show peak broadening due to the small sizes of the crystallites (Figure 4).

**Figure 5:**
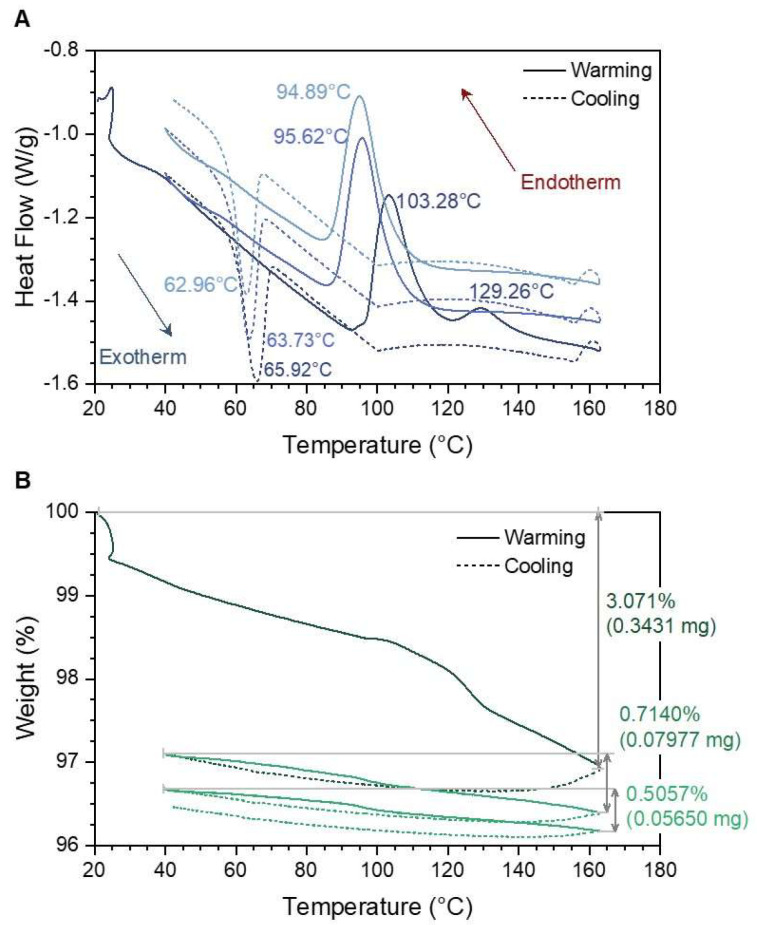
TGA and DSC data obtained for [Fe(Htrz)_2_(trz)](BF_4_)] as it was thermally cycled 3 times in an inert atmosphere. (**A**) DSC data on the phase changes as the sample is heated from room temperature to 160 °C for a total of 3 cycles. (**B**) TGA data on the weight changes that correspond to the 3 thermal cycles (marked 1, 2, and 3 next to heating and cooling transition temperatures). Thermal hysteresis can be seen in (**A**) between the exothermic (high to low spin state transition) and endothermic events (low to high spin transitions).

Thermal analysis. Thermal analysis of [Fe(Htrz)_2_(trz)](BF_4_)] nanoparticles was carried out, where both TGA and DSC data were collected between low and high spin states. Figure 5A shows DSC data corresponding to the thermal hysteresis of [Fe(Htrz)_2_(trz)](BF_4_)] in argon as it cycles three times from room temperature to 160 °C. A major change in heating events can be seen between the first and the second endothermic peaks, 103 °C and 95 °C, respectively. This drop in transition temperature is attributed to residual Fe(II) complex that remains in the high spin state even at room temperature [24,26,27]. Subsequent endothermic events following the second cycle decrease at a slow steady rate of ~1 °C per cycle. The exothermic events observed during the cooling cycles tend to follow the same trends as the second endothermic event onwards, decreasing at ~1 °C per cycle. Another endothermic event can be seen at 129 °C following the first cycle transition at 103 °C; this is associated with the loss of residual water following the transition from low to high spin.

Thermal analysis was also carried out in an oxygen-rich atmosphere (Figure S3A). The main effect relative to argon is that the temperature differences in the first and second endothermic peaks are much smaller. The argon sample shows a difference of almost 10 °C, whereas the sample in an oxygen-rich environment differed by 5 °C. Exothermic events occurred for the second and third thermal cycles, also displaying a slightly higher transition temperature when compared to the samples run in an inert atmosphere.

Weight loss data can be seen in Figure 5B for the inert sample and Figure S3B for the oxygenated sample. The only significant weight loss in both samples is seen after the first thermal event, which is around 3% and due to the loss of the surface water. As the sample experiences the remaining thermal cycles, the weight loss continues to decrease at lower rates. If the sample is exposed to temperature higher than 160 °C, sample degradation starts to occur in the first cycle.

**Figure 6:**
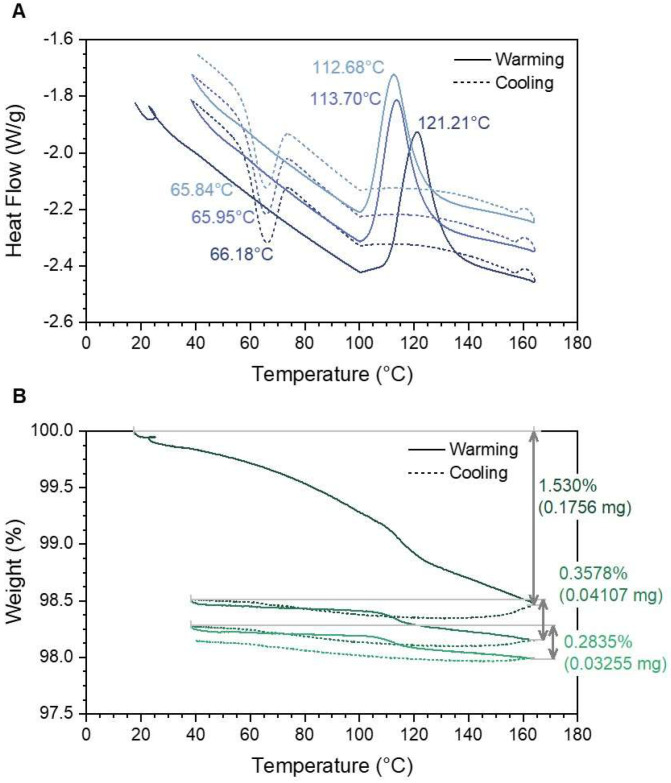
TGA and DSC data on [Fe(Htrz)_2_(trz)](BF_4_)] bulk samples that did not have any Tergitol NP9 present in the synthesis. (**A**) DSC data show higher temperatures for the transition from low spin to high spin when compared to nanoparticles in all three thermal cycles. (**B**) Weight loss for the bulk sample shows that less weight is lost in the three thermal cycles when compared to the nanoparticles.

Bulk [Fe(Htrz)_2_(trz)](BF_4_)] samples were also analyzed in an inert atmosphere and compared to the nanoparticle sample. Figure 6 shows both DSC and TGA data. Clear evidence is seen for higher transition temperatures when the sample undergoes the endothermic low spin to high spin change. Transitions from high spin to low spin states, however, occur at similar values, ~65 °C. Additionally, the percent weight loss seen in Figure 6B is smaller compared to [Fe(Htrz)_2_(trz)](BF_4_)] nanoparticles, likely due to the lower surface water relative to the high surface area nanoparticles. Of note was that endothermic events occurred at a much higher temperature compared to those samples with a surfactant. The weight loss data for all samples also indicate a slight weight gain as the sample transitions back to the low spin state. This weight gain is indicative of the possible formation of Fe_2_O_3_ on the surface of the compound. As the thermal cycles progress, a slight weight loss can be seen following the weight gain. Figure 6B shows a weight loss of 0.3578% for the second thermal cycle, which is less than that seen under argon due to the possible formation of iron oxide on the surface. Additional evidence to support iron oxide formation is observed when the sample is allowed to exceed 200 °C. Beyond this temperature, an orange residue is formed on the surface of the sample, similar to iron oxide. Table 1 summarizes the DSC and weight loss data of both nanoparticle samples and the bulk sample.

**Figure 7:**
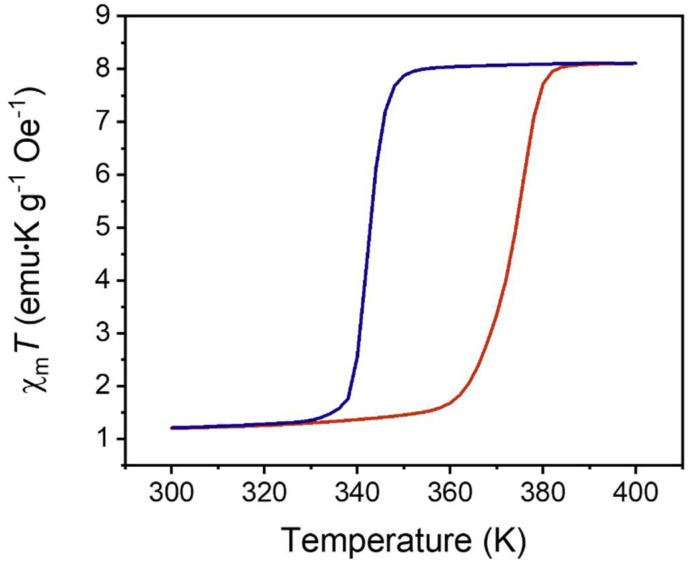
SQUID data obtained for [Fe(Htrz)_2_(trz)](BF_4_)] nanocubes using Tergitol-NP surfactant. Sample was held under a constant magnetic field of 1000 Oe. Initial transition from low to high spin state can be seen starting at 355 K. High to low spin transition occurred at 345 K which then stabilized at 335 K.

Magnetometry. Figure 7 shows the magnetic characterization using superconducting quantum interference device (SQUID) magnetometry of [Fe(Htrz)_2_(trz)](BF_4_)] nanoparticles at 1000 Oe as a function of temperature. Thermal hysteresis was observed, with a spin-transition onset of ~355 K upon heating. The transition temperature for the nanoparticles from low spin to high spin is much lower than what is seen in the bulk sample (370–380 K) [11] or other nanoparticles produced by different means (375–395 K) [27].

The transition from high to low spin seen on cooling occurs around 345 K. This is comparable to the exothermic transition temperatures seen from the DSC data (Figure 5A). The thermal hysteresis ranged from 335–375 K, with values above and below this range being the point at which the sample was predominately in either the high or low spin state, respectively. Field-dependent magnetometry data were also collected at different temperatures through vibrating sample magnetometry (VSM) (Figure S4). The magnetic moment as a function of field measured at room temperature (Figure S4A) did not show the expected diamagnetism. Instead, a paramagnetic signal was recorded with a reduced moment as compared to the moment recorded at elevated temperature (Figure S4B). This can be explained by the formation of Fe(III) in both systems. Figure S4C displays the VSM data acquired when the magnetic moment is presented as a function of the temperature. The magnetic field was held constant at 5000 Oe for the temperature gradient study, and output showed some similarities to the SQUID dataset.

## 3. Discussion

Surfactants are important components in the formation of nanoparticles [28]. The incorporation of Tergitol NP9 as a surfactant and ascorbic acid as a mild reducing agent in the synthesis of [Fe(Htrz)_2_(trz)](BF_4_)] effectively yielded nanocubes. Initial efforts without the use of a surfactant produced [Fe(Htrz)_2_(trz)](BF_4_)] crystallites of large dimensions. Figure S1A highlights a sample that was made without the use of any surfactant in the starting synthesis, and the dimensions of these particles averaged 476 nm in length and a width of 135 nm. The cylindrical shape of the particles is attributed to the [Fe(Htrz)_2_(trz)]^+^ chain structure [8]. These chains will propagate when suitable reaction conditions are met. Tergitol NP9 is a nonionic surfactant which has no net charge on its hydrophilic head group, therefore making it a very mild surfactant [29]. Due to this mild nature, it was a suitable candidate for the potential growth of the [Fe(Htrz)_2_(trz)](BF_4_)] nanoparticles. As seen in the initial trials using Tergitol NP9 with ascorbic acid, particles can be seen (Figure S1D) to have smaller dimensions than in the original synthesis. The amount of time allotted for the overall reaction also played a crucial part in the growth control. Leaving the particles to grow for 24-h with the use of Tergitol NP9 did reduce the dimension of the particles, but not by a significant amount. Looking at samples in Figure S1A,D, the difference in the length of the particles is only around 150 nm. The reaction time of 24-h was then modified to a very minimal amount to see at what extent the particles’ morphology would change. Figure S1B shows particles that were only allowed to grow for 5 min. The resulting nanoparticles still retained their rod-like shape, but the dimensions of these particles were not consistent, with particles ranging from 73 to 800 nm. Additionally, significant amounts of agglomeration could be seen throughout the sample. This agglomeration is due to a large excess of unreacted started materials that were not allowed sufficient time to react. The 24-h synthesis was then repeated, and the resulting particles maintained a length of around 300 nm, which would suggest that using those reaction conditions limited the growth to that length.

Further optimization was then conducted on the primary steps, leading to the final reaction. Initially, the iron (II) precursor was dissolved in the solvent before the addition of the triazole ligand and surfactant. The addition of the triazole ligand initiated the growth of the nanorods. Steps were taken to modify this stage, where both starting materials would be mixed in separate vials with the addition of the surfactant in both systems. The ascorbic acid, acting as a mild reducing agent, is then placed in the vial containing the iron (II) precursor. Both systems were mixed for 5 min before being combined. The resulting mixture, which was stirred for 5 min, produced particles with average lengths of 150 nm and widths of 56 nm (Figure 3). Tergitol NP9 controlled the growth of the particle, and further modification to the amount of surfactant used in the system was then studied. The synthesis required the use of a 4 g Tergitol NP9 (75% excess). This amount was adjusted to 80% (5 g) and 90% (10 g) to determine whether smaller particles were attainable. In theory, a higher concentration of surfactant should further limit the growth of the nanoparticle. In the case of the 80% fixed amount, particles grew to average lengths of 197 ± 44 nm, with the presence of heavy agglomeration. This is only a 50-nm difference from the samples that were made with a 75% amount. Reasons as to why this occurred could be that the higher concentrations of the surfactant led to more localized areas of growth as opposed to producing monodispersed growth. The 90% fixed amount showed potential signs of growth of smaller particles, but the agglomeration within the sample made it impossible to obtain any images via TEM, indicating that 90% surfactant was too high to allow effective formation of the nanoparticles.

Synthesis of [Fe(Htrz)_2_(trz)](BF_4_)] nanorods using Tergtitol-NP9 was established by Grosjean et al. [8,11,14]. Our modification of this approach allowed the formation of nanocubes. The resulting method included a 15-min stir at 80 °C of the separated vials, which had 4 g of surfactant and a 1-h reaction time at room temperature with constant stirring. This was then followed by a wash with ethyl acetate to halt the growth of the nanoparticles. The resultant nanoparticles were predominantly cubic in nature, with an average size of 49 nm (Figure 2). The incorporation of the heating phase in separate vials allowed for better mixing of the surfactant.

X-ray Diffraction. Figure S2 shows the simulated data obtained for [Fe(Htrz)_2_(trz)](BF_4_)] from the crystal structures reported by Grosjean et al. [8], highlighting the two spin states separately. The key feature that separates both is the overall pattern shift to the left when the transition occurs from low spin state (bottom) to high spin states (top). When looking at Figure 4, the observed XRD pattern for low spin state nanoparticles (top), broadening of the peaks is noticeable when comparing them to the simulated low spin data (bottom). Larger surface areas in the nanoparticle result in broadening of the peak of the observed XRD patterns. Dominant peaks at (2 0 0), (1 0 1), (3 0 1), (0 0 2), (4 1 0), (2 1 2), and (1 2 1) are seen in the observed XRD pattern for the nanoparticle. Grosjean et al. also reported XRD patterns for nanoparticles of similar size, but the definition of the peaks is not visible and very broad [8]. The observed XRD pattern in Figure 4 shows clear definition of the dominant peaks, which would indicate a more crystalline sample. When [Fe(Htrz)_2_(trz)](BF_4_)] transitions from the low spin to the high spin state, there is an expansion of the triazole ligand bonds which surround the Fe atom. The low spin state M–N (M = Fe, N = Nitrogen) bond length is 1.95 Å. This expansion adds 0.18 Å (based on thermal expansion) of the Fe–N bond length at high spin state [15,18,30]. The Fe–N–N bonds, which are the ligand bonds, have a bond angle that ranges from 120° to 128°, depending on the spin state. The overall volume expansion from low spin state to high spin states is 8.4%. At high spin states, due to the natural flexibility of the triazole ligands, the expansion can remain stable as long as the thermal threshold is not exceeded (above 160 °C for [Fe(Htrz)_2_(trz)](BF_4_)]).

Thermal Analysis. Thermal analysis was conducted on [Fe(Htrz)_2_(trz)](BF_4_)] to determine the spin transition points of the newly acquired nanocubes. DSC data conducted in an inert atmosphere on bulk [Fe(Htrz)_2_(trz)](BF_4_)] without the use of Tergitol NP9 showed the transition point to high spin state as being around 121 °C (Figure 6). This was then followed with a transition back to the low spin state at 66 °C. These transitions concur with already established bulk data [11,12,15]. However, additional thermal cycles were implemented on the system to determine the stability of the compound. A total of three thermal cycles were completed, and differences were seen between the first and consecutive results. Figure 6 depicts a difference of almost 10 °C between the first and second endothermic events (high spin transitions). The second and third endothermic events were steadily decreasing at a rate of 1 °C per cycle. Exothermic events (low spin transitions) remained stable and constantly decreased at 1 °C per cycle. Variations in these transitions in bulk sample can be seen in the nanoparticle system that contained Tergitol NP9. Figure 5A shows that the first transition from low to high spin states occurs at a lower temperature, 103.28 °C. This was then followed by a difference of 8 °C between the first and second endothermic event, which eventually became a steady decrease in the following cycle of 1 °C. A similar case was seen in the exothermic events which occurred at 65 °C. Clear evidence is seen of lower transition temperatures when the nanoparticles are compared to the bulk system. Differences in the thermal hysteresis width between the bulk versus the nanoparticles were 18 °C. Smaller transitions along with smaller thermal hysteresis are observed within the nanoparticle systems. Coronado et al. observed that some Fe(II) complexes remained in the high spin state after the first thermal transition from low spin to high spin [24]. This can produce a high surface area effect in the nanoparticles, which results in a preferential coordination of the Fe atom to the high spin state, even at room temperature. This results in smaller amounts of Fe(II) that are in low spin state and high spin state Fe(II) at room temperature, which reduces the thermal hysteresis in the following cycles. Both Tissot et al. and Coronado et al. showed evidence that 20% of the Fe(II) complexes remained in the high spin state when conducting further thermal cycles [24,31]. This explains the difference between the first and second endothermic peaks in both the inert and oxygenated environment for both nanoparticle and bulk systems. Nanoparticle systems show a decrease in the hysteresis loop when compared to bulk material. The effects of this trapped high spin state Fe(II) are therefore less in the nanoparticle system than in bulk material. Thermal hysteresis for nanoparticles (Figure 5A) was a total 37 °C, whereas the bulk system (Figure 6A) without Tergitol NP9 was 55 °C.

Weight loss associated with the nanoparticle system for the first transition from low spin to high spin, Figure 5B, shows a weight loss primarily due to surface water of only 3% of the overall weight of the sample. Dîrtu et al. have observed similar behavior in an amino–ester derivative [32]. There is also a small weight gain as the temperature transitions back down to room temperature of less than 0.5%, which is seen in each thermal cycle. This weight gain can be attributed to the formation of iron oxide on the surface of the sample. In a separate study, the sample was kept constant at 80 °C for 60 min to determine if further weight gain occurred. The resulting data from that study showed that the sample gained weight relatively slowly as it remained at 80 °C for the allotted time. Allowing the sample to remain at this temperature on the cool down results in the formation of iron oxide on the surface of the nanoparticles. This behavior was also seen in the bulk sample, but with a relatively smaller weight gain when compared to the nanoparticles. Nanoparticles have larger surface areas to allow for the formation of more iron oxide on the surface as compared to the bulk samples. The slight weight gain then occurs when the sample repeats the second thermal cycle; weight change is possibly due to loss of water or organic substituents. This behavior of weight gain and weight loss is seen consistently as the sample continues the remaining thermal cycles. Further evidence to support the formation of iron oxide is shown when the sample is allowed to exceed 200 °C. Past these temperatures, the iron complexes more extensively convert to iron oxide as indicated by the observation of a more prominent appearance of residual surface orange/red color on the sample. The following weight changes for the other two thermal cycles are <1%, which would indicate that the sample loses weight at an extremely slow rate as the thermal events takes place. Figure S3A shows the DSC data for a Tergitol NP9 sample that was conducted in an oxygenated environment. The only significant differences between this sample and the inert sample were that the thermal transitions for both low spin to high spin and vice versa occurred at a slightly higher temperature. Higher transition temperatures in the oxygenated system would indicate that the surrounding oxygen helped to stabilize the complex, thus preventing the transition from occurring earlier. The sample reacted with the surrounding oxygen to form more iron oxide on the surface as heating takes place, thus prolonging the transitions from low spin to high spin. Looking at the weight loss data in Figure S3B, the weight gain is slightly larger than the sample conducted in an inert atmosphere. Although the weight loss is still <1% for the overall system in the second and third endothermic events, the oxygenated system lost less weight when compared to the inert system. Relating the bulk data to the nanoparticle data, weight loss on the whole is significantly less in the bulk system. The surface area of the nanoparticles is much larger than in the bulk system; therefore, the amount of iron oxide formed on the surface of the bulk system is significantly less.

Magnetism. Figure S5 is a diagram showing the two energy levels that are obtained when the triazole ligand forms a coordinate bond with Fe^2+^. The six electrons that are in the d orbitals of Fe^2+^ are separated into low spin states or high spin states depending on the stimuli to which they are exposed [8,16]. In the high spin state, the energy gap between the e_g_ and t_2g_ level is small enough to allow two unpaired electrons to occupy the higher energy levels (Figure S5A). According to Hund’s rule and Aufbau principles, electrons must populate the lower energy orbital first. The t_2g_ energy levels for the low spin state would be occupied by all six electrons, because the amount of energy required to pair the electron is much lower than that to populate the higher orbitals. Therefore, with no unpaired electrons, diamagnetic behavior is seen, as there are no unpaired electrons to interact with a magnetic field. In the case of the high spin state, the energy difference between the e_g_ and t_2g_ orbitals is small enough to allow electrons to populate the high orbitals as opposed to pairing. This produces two unpaired electrons in both the e_g_ and two unpaired electrons in the t_2g_ levels, producing a sum spin of two which can respond to a magnetic field, producing paramagnetic characteristics and magnetic hysteresis. The magnetic moment as a function of temperature can be seen in Figure 7, where [Fe(Htrz)_2_(trz)](BF_4_)] is at a constant field of 1000 Oe. As the temperature approaches 355 K, the transition to high spin state is initiated and comes to completion at 375 K. This is lower than the transitions reported in bulk systems [8,28,31]. The response to the magnetic field is also seen as it increases from 1.2 χ_m_ to a max of 8 χ_m_ in the transition from low spin to high spin state. When transitioning back to the low spin state, a further increase is seen in the magnetic response to around 8 χ_m_ before decreasing back to 1.1 χ_m_. Despite the relatively small change in the magnetic response as the temperature increases, it was still possible to obtain temperature-dependent hysteresis for the [Fe(Htrz)_2_(trz)](BF_4_)] nanocubes. Overall, the temperature-dependent hysteresis for the SQUID results had a width of 45 K with initial low spin to high starting at 335 K and high spin to low spin at 350 K.

To obtain comparative data for the SCO nanoparticles, VSM analysis was then implemented to determine whether the thermal hysteresis observed would be the same as that found in the SQUID. Figure S4C displays the VSM data obtained for the same sample, but at a constant magnetic field of 5000 Oe. Due to the lower sensitivity of the VSM compared to SQUID–VSM, a higher magnetic field had to be used to produce a quantifiable magnetic response from the nanoparticles. The response to the magnetic field occurs at 393 K with a magnetic moment of 5.85 × 10^−4^ emu, which stabilized at 430 K with moments of 2.22 × 10^−3^ emu. High spin to low spin transition occurred at 360 K with a response of 2.49 × 10^−3^ emu followed by stabilization at 340 K with a response of 5.00 × 10^−4^ emu. The resulting thermal hysteresis for the VSM (Figure S4C) was 90 K in width, which was larger than the previous SQUID findings by 50 K. SQUID measurements showed an initial transition to high spin state around 355 K. At this temperature, no indication of a transition was present in the VSM analysis. The difference between the magnetic moment at the end of the cooling cycle and at the start of the heating cycles is the larger of the VSM data when compared to the SQUID. SQUID magnetic moments for this difference are 5%, while VSM produced a difference of 18%. Table 2 summarizes and compares the data between the transitions for the low spin states and high spin states of the thermal and magnetic analysis.

VSM analysis was also conducted on the individual spin states. Figure S4A depicts the magnetic moments of the nanoparticles at room temperature. Paramagnetic behavior is seen in Figure S4B, which is expected at the high spin states. However, in the low spin state, a diamagnetic response should have been observed in the data but, as seen in Figure S4A, this was not the case. Paramagnetic behavior was observed at room temperature with no characteristics of diamagnetic nature. The formation of Fe(III) in the system caused this paramagnetic characteristic to be seen in the low spin state data of the VSM [24,31]. The nature of Fe^3+^ allows it to have five electrons in its lower t_2g_ orbital. Four of the five electrons pair up, leaving only one unpaired electron, which can have a magnetic response even at room temperature. This is the reason why the paramagnetic behavior was observed for samples conducted at room temperature on the VSM

## 4. Materials and Methods

Materials. Tergitol NP9 (non-ionic poly(oxyethylene), surfactant), Iron (II) tetrafluoroborate hexahydrate (Fe(BF_4_)_2_·6H_2_O, 97%), 1,2,4-triazole (98%), L-ascorbic acid (ACS reagent, 99%), and ethyl acetate (ACS reagent, 99.5%) were purchased from Sigma Aldrich (St. Louis, Mo, USA). Distilled water was obtained via reverse osmosis.

Synthesis of [Fe(Htrz)_2_(trz)](BF_4_)] nanoparticles. Synthetic procedures were adapted from a reverse micelle method described in literature [8]. Two micellar solutions were prepared separately, with a surfactant amount fixed at 75% excess of the solvent. In the first solution, Fe(BF_4_)_2_·6H_2_O (3 mmol) and ascorbic acid (10 mg) were added to distilled water (1 mL). The solution was stirred until the compounds dissolved; this was then followed by the addition of Tergitol NP9 surfactant (4 g). In the second solution, 1,2,4-triazole (9 mmol) was added to distilled water (1 mL) and stirred until all compound dissolved. Tergitol NP9 (4 g) was then added to the second solution after stirring. The separate micelle solutions were stirred and heated to 80 °C for 15 min. The solutions were quickly combined, and the mixture was stirred for 1 h at room temperature. The reaction was stopped by adding ethyl acetate (particle sedimentation). The nanoparticles appeared as a violet precipitate obtained after the first wash/separation and centrifugation (13,000 rpm) with ethyl acetate (10 min). Wash/separation steps were repeated three times under the same conditions. Particles of different sizes were created by varying the amount of ascorbic acid, preparation time, and reactant concentrations (Table S1).

## 5. Conclusions

The present study provides new data on the transitions of spin cross over compound [Fe(Htrz)_2_(trz)](BF_4_)] nanoparticles. With the use of a mild nonionic surfactant, Tergitol NP9, it was possible to synthesize [Fe(Htrz)_2_(trz)](BF_4_)] particles with dimensions less than 100 nm in length. Careful reaction conditions must be met in order to obtain a sample of [Fe(Htrz)_2_(trz)](BF_4_)] with resulting nanocubes of 49 ± 4 nm in size. Observed XRD patterns matched the simulated data for the low spin state complex. Thermal hysteresis analysis determined that the nanoparticle system underwent transitions from low spin state to high spin state at lower temperature when compared to bulk system and other particles of the same size. Trapped high spin state complexes in the low spin state after the first transition led to the decrease in the width of the hysteresis. Magnetic hysteresis data also agreed with the thermal findings on the spin transition points of the nanoparticles. SQUID and DSC data displayed close agreements with both high spin and low spin transitions of the nanoparticles. These transition points were lower than the data already reported for bulk and other nanoparticles of [Fe(Htrz)_2_(trz)](BF_4_)]. By further modification to reduce to size of the particles and, thereby, reduce transition temperature, it is possible to develop nanoparticles with ideal characteristics for applications such as switches and memory storage.

## Figures and Tables

**Table 1:** Summary of all the DSC and TGA weight loss data obtained for nanoparticles (Samples 1 and 2) and bulk samples (Sample 3) of [Fe(Htrz)_2_(trz)](BF_4_)]. Transitions from low spin states to high spin state in nanoparticle samples occur at lower temperatures when compared to the bulk material.

Sample Number	1st Thermal Cycle			2nd Thermal Cycle			3rd Thermal Cycle			Atm.
	Endotherm (LS to HS) (°C)	Exotherm (HS to LS) (°C)	Weight Loss (%)	Endotherm (LS to HS) (°C)	Exotherm (HS to LS) (°C)	Weight Loss (%)	Endotherm (LS to HS) (°C)	Exotherm (HS to LS) (°C)	Weight Loss (%)	
1	103.28	65.92	3.07	95.62	63.73	0.714	94.89	62.96	0.506	Ar
Thermal Hysteresis	37 °C			32 °C			32 °C			
2	103.08	66.29	2.95	98.13	65.38	0.623	97.96	65.21	0.499	Ar:O_2_ (50:50)
Thermal Hysteresis	37 °C			33 °C			33 °C			
3	121.21	66.18	1.53	113.70	65.95	0.358	112.68	65.84	0.283	Ar
Thermal Hysteresis	55 °C			48 °C			47 °C			

**Table 2:** Summary and comparison of the data collected for the transitions between low spin state to high spin state and vice versa, between three datasets collected for [Fe(Htrz)_2_(trz)](BF_4_)] nanocubes.

Data	HS Transition	LS Transition	Hysteresis Width
DSC	376 K	338 K	37 K
SQUID	375 K	330 K	45 K
VSM	430 K	335 K	90 K

## Data Availability

Data are contained within the article and Supplementary Material.

## Supplementary Materials

The following supporting information can be downloaded at. Table S1: Table of conditions for the production of [Fe(Htrz)2(trz)](BF4)] nanocubes, Figure S1: TEM images of various [Fe(Htrz)2(trz)](BF4)] nanoparticles, Figure S2: Simulated XRD patterns for both low spin and high spin states, Figure S3: TGA–DSC data obtained for [Fe(Htrz)2(trz)](BF4)] (Reaction 9), Figure S4: VSM datasets of [Fe(Htrz)2(trz)](BF4)] nanoparticles studied under a constant magnetic field of 5000 Oe, Figure S5: Energy level difference d-orbital diagram between the high and low spin states.

## Sample Availability

Authors will consult researchers on the synthesis of these compounds and under special circumstances, provide limited amounts of prepared nanoparticles.
