# Correction: Blanco et al. Synthesis and Characterization of [Fe(Htrz)_2_(trz)](BF_4_)] Nanocubes. *Molecules* 2022, *27*, 1213

**DOI:** 10.3390/molecules29010266

**Published:** 2024-01-04

**Authors:** Alexis A. Blanco, Daniel J. Adams, Jason D. Azoulay, Leonard Spinu, John B. Wiley

**Affiliations:** 1 Departments of Chemistry and Physics, Advanced Materials Research Institute, University of New Orleans, New Orleans, LA 70148, USA; aablanc1@my.uno.edu (A.A.B.); lspinu@uno.edu (L.S.); 2 School of Polymer Science and Engineering, The University of Southern Mississippi, Hattiesburg, MS 39406, USA; daniel.adams@usm.edu (D.J.A.); jason.azoulay@usm.edu (J.D.A.)

After publication of the paper [1], the authors were made aware of the thesis of Celine Etrillard (C. Etrillard, PhD Thesis, University of Bordeaux, 2011), which originated the use of the micelle method described in reference 8 for the preparation of a variety of nanoparticles, including those with cube-like geometries. Further, the authors made an error in the references in the last sentence of the Introduction.

## Original: 

These spin transitions occur at lower temperatures when compared to bulk analogs [8,11,14] and other nanoparticles made with different starting materials [23,24].

## Corrected: 

These spin transitions are known to occur at lower temperatures [8,11] when compared to bulk analogs and other nanoparticles made with different starting materials [23,24].
