# Supplementary material for: Synthesis and Characterization of [Fe(Htrz)2(trz)](BF4)] Nanocubes
Source: Molecules. 2022 Feb 11;27(4):1213. doi: 10.3390/molecules27041213 (PMC8874602; doi:10.3390/molecules27041213)
Supplement: Supplementary file 1 [file molecules-27-01213-s001.zip › molecules-1564930-supplementary.pdf]

Synthesis and Characterization of [Fe(Htrz)$_2$(trz)](BF$_4$)] Nanocubes

Alexis A. Blanco,[1] Daniel J. Adams,[2] Jason D. Azoulay,[2] Leonard Spinu,[1] and John B. Wiley*,[1]

[1]Department of Chemistry and Advance Materials Research Institute, University of New Orleans, New Orleans, Louisiana 70148, United States

[2]School of Polymer Science and Engineering, The University of Southern Mississippi, Hattiesburg, Mississippi 39406, United States

Supporting Information

Tables:

Table S1. Table of conditions for the production of [Fe(Htrz)$_2$(trz)](BF$_4$)] nanocubes

Figures:

Figure S1. TEM images of various [Fe(Htrz)$_2$(trz)](BF$_4$)] nanoparticles.

Figure S2. Simulated XRD patterns for both low spin high spin states

Figure S3. TGA-DSC data obtained for [Fe(Htrz)$_2$(trz)](BF$_4$)] (Reaction 9).

Figure S4. VSM data sets of [Fe(Htrz)$_2$(trz)](BF$_4$)] nanoparticles studied under a constant magnetic field of 5000 Oe.

Figure S5. Energy level difference *d*-orbital diagram between the high and low spin states.

Table S1. Table showing the systematic pathway that was developed to determine the optimal method of the production of [Fe(Htrz)$_2$(trz)](BF$_4$)] nanocubes. Reaction 9 was used for the synthesis of the nanoparticles seen in Figure 2. *Nanoparticles formed were heavily agglomerated with amorphous morphology.

Rx #	Fe(BF$_4$)$_2$ (g)	Triazole (g)	Solvent (mL)	Tergitol NP9 (g)	Ascorbic Acid (mg)	Separate Stir Time (mins.)	Separate Stir Temp. (°C)	Reaction Time (mins.)	Nanoparticle Length (nm)
1	0.675	0.414	1.6	0	0	0	N/A	1440	476 (rod)
2	0.675	0.414	1.0	4	50	0	N/A	1440	309 (rod)
3	0.360	0.220	1.0	4	10	0	N/A	5	73-800
4	0.360	0.220	1.0	4	10	5	25	1440	200 (rod)
5	0.360	0.220	1.0	4	10	5	25	5	150 (rod)
6	0.360	0.220	1.0	4	3	5	25	5	N/A*
7	0.360	0.220	1.0	5	10	5	25	5	200 (rod)
8	0.360	0.220	1.0	10	10	5	25	5	N/A*
9	1.08	0.660	1.0	4	5	15	80	60	49 (cubes)

Figures

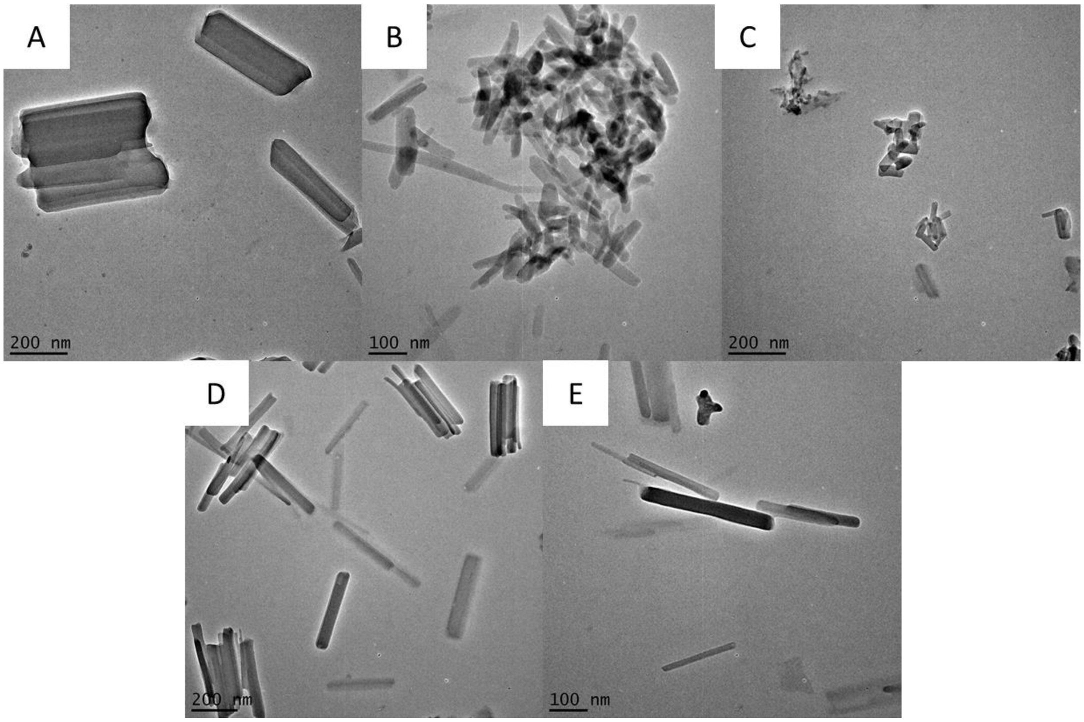

Figure S1. TEM images of [Fe(Htrz)$_2$(trz)](BF$_4$)] nanoparticles from various reactions in Table S1: (a) Reaction 1 (b) Reaction 3 (c) Reaction 6 (d) Reaction 2 and (e) Reaction 4. Heavy agglomeration can be found in (b) due to the short time allotted for the reaction time. More defined nanoparticles can be seen in (d) and (e). Amorphous particles can be seen in (c) where 3 mg of ascorbic acid was used.

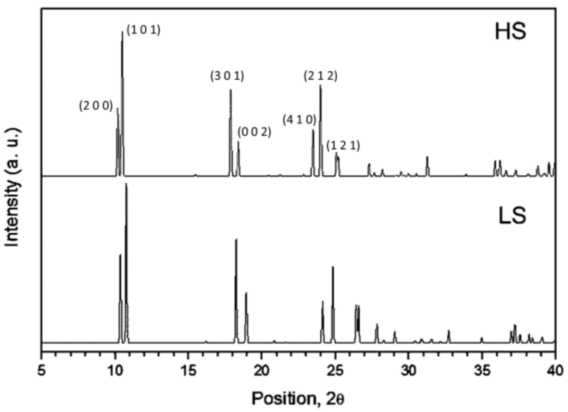

Figure S2. Simulated XRD pattern that shows both the low spin state (bottom) and the high spin state (top). The difference between the low spin state and high spin state is a left shift when the transition from low to high spin occurs.

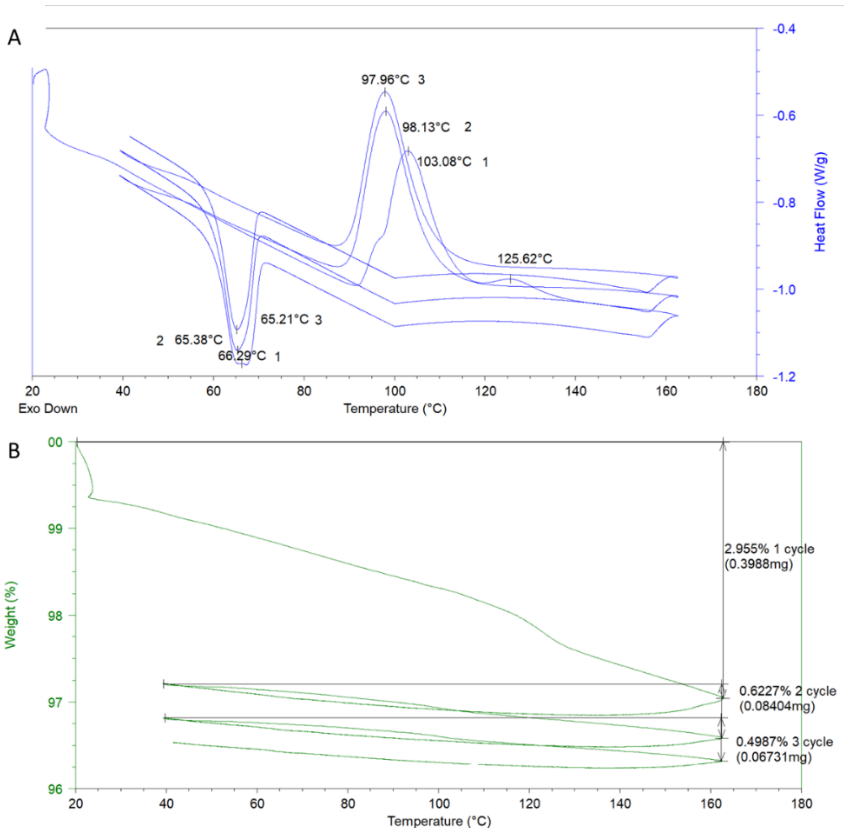

Figure S3. TGA-DSC data obtained for [Fe(Htrz)$_2$(trz)](BF$_4$)] as it was thermally cycled three times in an atmosphere of 50% argon and 50% oxygen. (a) DSC data on the phase changes as the sample is heated from room temperature to 160 ºC for a total of 3 cycles. (b) TGA data on the weight changes that correspond to the 3 thermal cycles. Thermal hysteresis can be seen in (a) between the exothermic and endothermic events.

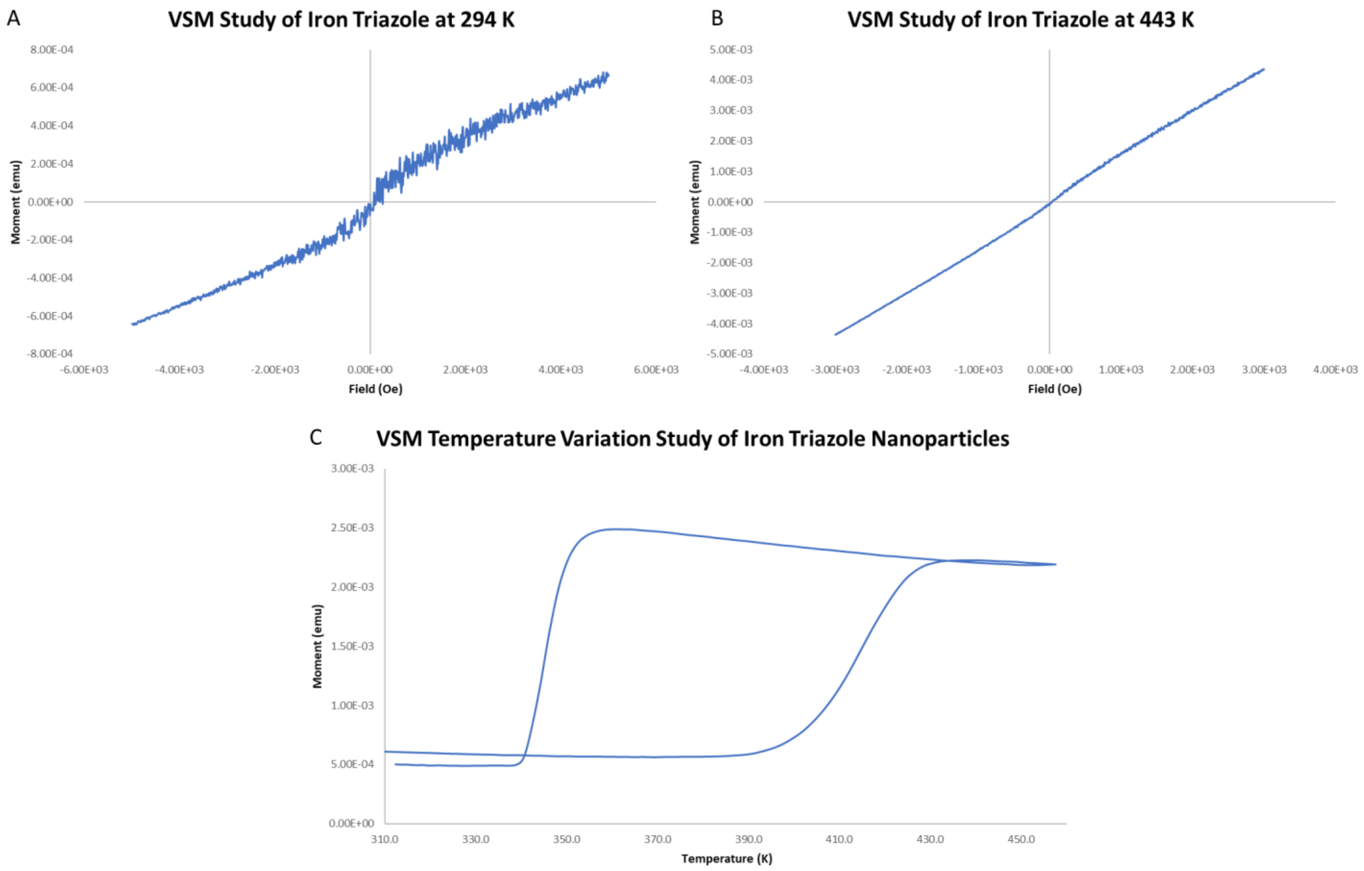

Figure S4. VSM data sets of $[Fe(Htrz)_2(trz)](BF_4)]$ nanoparticles studied under a constant magnetic field of 5000 Oe. (a) $[Fe(Htrz)_2(trz)](BF_4)]$ nanoparticles studied at room temperature. This system should have shown diamagnetic behavior but instead displayed paramagnetic characteristic. This could be due to possible Fe(III) in the compound or trapped high spin state Fe(II) complexes. (b) $[Fe(Htrz)_2(trz)](BF_4)]$ nanoparticles studied at a constant 443 K. Paramagnetic characteristics can be seen in the data obtained. (c) Temperature variation study of $[Fe(Htrz)_2(trz)](BF_4)]$ 294 K to 443 K. Magnetic hysteresis is seen with a width of 90 K.

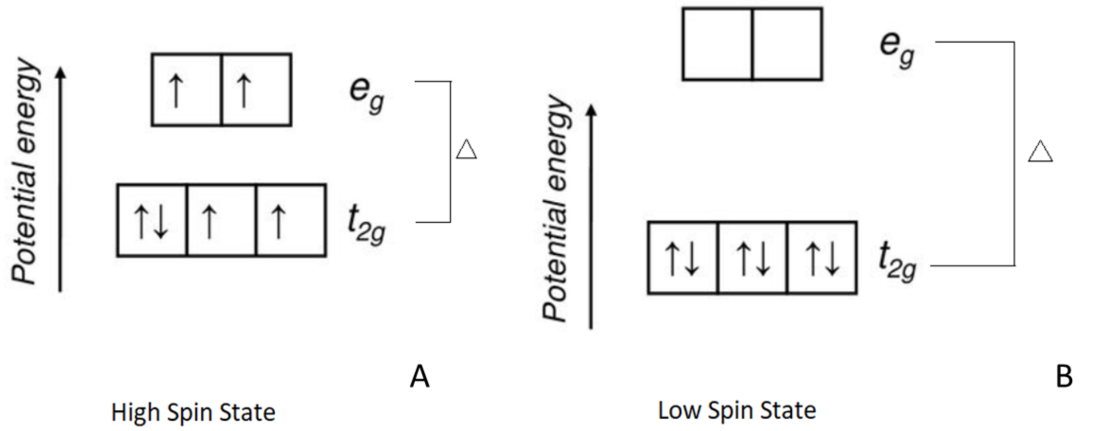

Figure S5. Energy level difference between the high spin states (a) and low spin states (b) of the d^6 Fe^{2+} core of $[Fe(Htrz)_2(trz)](BF_4)]$. (a) The difference in energy level between the e_g and t_{2g} in the high spin state allows the lone pairs to occupy the empty orbitals. This produces a sum of spin of 2 due to the 4 unpaired electrons. This state is known as the high spin state due to the larger amount of total spin than can respond to a magnetic source when compared to the low spin state. Typical paramagnetic characteristics can be noticed from species in the high spin state. (b) Low spin d^6 state compounds display diamagnetic behavior due to lack of unpaired electron. The energy level is too high between the e_g and t_{2g} levels to allow electrons to occupy the higher energy states.
